# How can the transition from office to telework be managed? The impact of tasks and workplace suitability on collaboration and work performance

**DOI:** 10.3389/fpsyg.2022.987530

**Published:** 2022-10-26

**Authors:** Tobias Müller, Florian Schuberth, Micha Bergsiek, Jörg Henseler

**Affiliations:** ^1^Faculty of Engineering Technology, Chair of Product-Market Relations, University of Twente, Enschede, Netherlands; ^2^Fachhochschule der Wirtschaft, University of Applied Sciences, Paderborn, Germany; ^3^Nova Information Management School, Universidade Nova de Lisboa, Lisbon, Portugal

**Keywords:** COVID-19, partial least squares path modeling (PLS-PM), emergent variables, formed concept, telework, composite modeling, PLSc

## Abstract

COVID-19 made evident the need for workplace digital transformation due to a rapid transition from office to remote work. Therefore, employers must make telework suitable for office workers who suddenly became permanent teleworkers. By using partial least squares path modeling, this article suggests the defining of telework tasks suitability and of telework workplace suitability by performing an empirical study with 691 employees who had experienced a rapid transition from office work to remote work during the pandemic. Both telework tasks suitability and telework workplace suitability are found to have a positive relationship with collaboration and work performance. Employers should therefore especially focus on communication technology when expecting employees work from home to improve work performance and enable collaboration to prevent them from feeling isolated. This study is the first to define telework tasks suitability and workplace suitability for enabling collaboration and improving work performance of teleworkers after an enforced transition from office working to teleworking.

## 1. Introduction

Before the outbreak of COVID-19, telework was a “luxury for the relatively affluent” (Desilver, 2021) whose actual job tasks allow spending a small part of the working week from home (Parent-Thirion et al., 2017). Such workers tend to be rather experienced in telework, apply theoretical and abstract knowledge (Frenkel et al., 1995) and have also benefited from increasing digitalization in the workplace, i.e., accessing work remotely from anywhere and at any time. Conversely, there are those who have only little or no experience of telework, who typically rely more on contextual knowledge and less on intellectual and creative knowledge (Frenkel et al., 1995). For many of such millions of workers, telecommuting had not been a widely used and had been almost inaccessible due to job status and the nature of their tasks (Kossek and Lautsch, 2018). COVID-19 totally changed all that almost overnight. Since governments enforced lockdown measures and social distancing, traditional workplaces underwent sudden shifts toward remote working—“the new normal”—regardless of whether job tasks actually suit telework. Hence, the maxim that “[j]ob suitability reigns high among traits considered indicative of which employees are eligible for telework” (Bailey and Kurland, 2002, p. 386) suddenly became obsolete. Instead, managers were forced to make many decisions in very short periods of time about who should work from the office or from home, how and where people could be moved into digital space, what the new priorities are, and how such priorities can best be communicated to employees (Caligiuri et al., 2020). Challenges, such as the ability to effectively use technology, to work in social isolation, and to work at home while often undertaking caring duties, have had sudden but often long-lasting impacts on workers who were forced by the pandemic to telework (Zacher and Rudolph, 2021). As a result, the pandemic has brought to surface the need for workplace digital transformation (Branicki, 2020).

Consequently, a systematic understanding of the potentially changed nature of work tasks and of the workplace in the different context is required (Wang et al., 2020). Against this background, scientists and practitioners demand answers as to how to enable telework tasks suitability, i.e., the content and organization of work tasks, activities, relationships, and responsibilities (Parker, 2014), and how to define telework workplace suitability, i.e., the work environment and the necessary equipment. More concretely, research needs to examine how remote working tasks and the remote workplace should be defined to enable teleworkers to collaborate with their stakeholders to overcome the threat of being isolated when working remotely; and maintain a high level of work performance. Such research should pay particular attention to those who have involuntarily experienced the rapid transition from office work to remote work and to those who have experienced teleworking for the first time during the pandemic (Caligiuri et al., 2020), i.e., office workers who suddenly became (permanent) teleworkers. Bridging this gap in the literature would not only help new teleworkers to successfully fulfill their remote job tasks, but also help experienced teleworkers to improve their remote workplaces. Moreover, knowledge in this field is relevant for supervisors, as many of those had to make quick decisions about how to enable inexperienced teleworkers to successfully fulfill their job tasks in a remote setting. Reliable knowledge for supervisors about the suitability of the digital work and workplace would be relevant to help them assign suitable tasks for remote working and to support teleworkers who might lack of a suitable telework environment or work equipment.

This study draws on the Job Demands-Resources (JD-R) model (Demerouti et al., 2001). More specifically, we adopt the framework proposed by Bakker and Xanthopoulou (2013), that studies the impact of job resources on creativity and charismatic leadership in the telework context. In the telework context, telework tasks and workplace suitability as job resources are crucial success factors for corporations and their employees in complying with the new normal and to succeed in the transition from a physical work environment to a more digital work environment (e.g., Bailey and Kurland, 2002; Nakrošienė et al., 2019). Similarly, it is widely reported in the literature that collaboration improves work performance (e.g., Wageman and Baker, 1997; Van der Vegt and Van de Vliert, 2016). Therefore, we postulate that telework tasks suitability, telework workplace suitability, and collaboration have a positive relationship with work performance. In doing so, we especially focus on those employees who have experienced a rapid enforced transition from office to remote work and by the majority worked remotely for the first time in the pandemic.

The remainder of this paper is structured as follows. In Section 2, we introduce our theoretical framework and develop our hypotheses. In Section 3, we present the methodological part, i.e., sample and data, concept operationalization, and model estimation. Next, the results of our study are presented in Section 4. Finally, in Section 5, we discuss our findings and offer theoretical and practical suggestions to improve the experience and effectiveness of employees engaged in remote work.

## 2. Theory, conceptualization, and hypothesis development

In our study, we adapt the Job Demands-Resources (JD-R) model. Originally, that model was proposed to explain the impact of job resources such as autonomy, social support, performance feedback, opportunities for professional development, and of job demands leading to burnout, i.e., exhaustion and disengagement from work (Demerouti et al., 2001; Bakker and Demerouti, 2007, 2017). Subsequently, the JD-R model was applied to other contexts such as work stress in occupational health (Demerouti et al., 2001; Britt et al., 2021) and project leadership (Lattrich and Büttgen, 2020).

**Figure 1:**
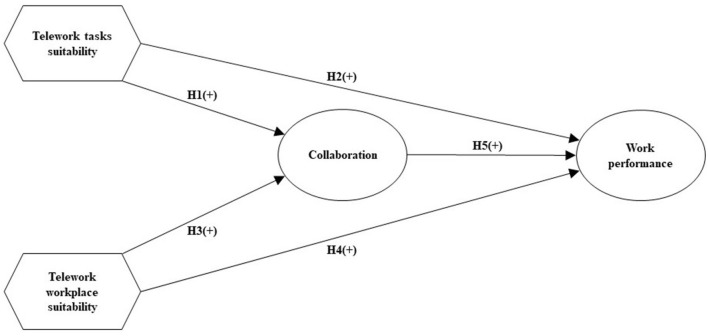
Conceptual model.

In this study, we rely on the framework proposed by Bakker and Xanthopoulou (2013) and apply it to the telework context. While the JD-R model is originally concerned with both job resources and job demands, in their study Bakker and Xanthopoulou (2013) focused solely on job resources and their indirect impact on creativity and charismatic leadership. In our study, we investigate the effects of job resources in the form of telework tasks suitability and telework workplace suitability on collaboration and work performance. It is widely reported in the literature that job resources may promote a motivational process that can lead to a high level of work performance (Bakker and Demerouti, 2007, 2017). Moreover, telework tasks and telework workplaces can be regarded as suitable if they overcome drawbacks of telework such as feeling isolated (e.g., Cooper and Kurland, 2002; Golden et al., 2008), which may result in collaboration among teleworkers. Collaboration, social integration, or getting together frequently to perform tasks can reduce the impact of demographic differences (Elsass and Graves, 2001) and has a strong, positive impact on task performance (Harrison et al., 2002), i.e., work performance. Our conceptual model is shown in Figure 1.

### 2.1. Conceptualization

In our study, we examine telework tasks suitability, telework workplace suitability, collaboration and work performance. In this section, we elaborate on how these concepts have been conceptualized.

#### 2.1.1. Telework tasks suitability

To protect employees from physical health risks associated with COVID-19, many office workers suddenly had to become teleworkers. This enforced transition from office to teleworking posed challenges for both employees and managers, since extensive teleworking can damage coworker relationships (Fonner and Roloff, 2010). In addition, if teleworkers feel excluded and miss their former office interactions, reductions in visibility and decreased career development opportunities are likely (Duxbury et al., 1998; Mann et al., 2000; Harpaz, 2002; Felstead et al., 2003; Harris, 2003; McDonald et al., 2008; Grant et al., 2013). However, if teleworkers experience a sense of autonomy (Gajendran and Harrison, 2007), higher feelings of control, and flexibility (Huws et al., 1990; Standen et al., 1999; Maruyama and Tietze, 2012), then it is more likely that they will appreciate teleworking. In fact, high work performance is among the most commonly cited positive outcomes of telework (Khanna and New, 2008) if telework is suitable.

Social, organizational or physical aspects of a job can be qualified as job resources if they are functional in achieving work goals and/or stimulating personal growth, learning, and development (Bakker and Xanthopoulou, 2013). Considering the job resources provided by the JD-R model, to achieve work goals and to stimulate personal development during enforced telework, the concept telework tasks suitability can be defined as a composition of social and organizational aspects to cover the job resources autonomy, social support, performance feedback, and opportunities for professional development.

Telework tasks suitability covers the need for autonomy by facilitating familiar work tasks in an enforced telework environment; familiar work tasks enable a high level of autonomy (Xanthopoulou et al., 2007). Additionally, it covers the relevance of social support, which is particularly important as a negativity buffer, helping teleworkers to cope with stress and to focus on tasks (Bavik et al., 2020; Wang et al., 2020). Moreover, it covers organizational social support, which is pivotal to successfully managing the boundary between work and personal roles as one of the key challenges inherent in telework (Golden et al., 2006). Specifically, telework tasks suitability has to ensure that remote workers (a) stay connected to their stakeholders, i.e., colleagues, supervisors, and customers (Mathieu et al., 2000; Mann and Holdsworth, 2003; Grant et al., 2013; Kwahk and Park, 2016) to obtain social support and to stimulate performance feedback; (b) can process their daily work routines (Huws et al., 1990; Standen et al., 1999; Maruyama and Tietze, 2012) and, as a result, achieve high levels of autonomy; and (c) can address their need for professional growth by assigning, conducting and consulting about (new) projects, concepts, and topics (Deci et al., 2017; Caligiuri et al., 2020).

#### 2.1.2. Telework workplace suitability

The unexpected transition from full-time office work to full-time telework during lockdown challenged all members of teleworkers' households who found themselves together at home almost permanently. Limited space at home and permanent distractions made it difficult to concentrate, affecting employees' ability to empathize with others, to consider plausible alternatives, to remain open-minded, to engage in cognitively challenging tasks and to expand their experiences in order to learn and grow (Caligiuri et al., 2020). Many households lacked suitable working equipment, e.g., notebooks, tablets, to connect all household members remotely. Consequently, in an inappropriate remote working place, employees struggled with their autonomy or ability to make decisions and to exercise a degree of discretion over the work to be accomplished; hence, they lost autonomy and control over their work (Hackman and Oldham, 1980; Spector, 1986). However, if the remote workplace was suitably equipped according to the needs of teleworkers, the ability to concentrate was higher and the need for recovery was lower on home working days than on office working days (Mann et al., 2000; Nardi and Whittaker, 2002; Konradt et al., 2003; Biron and van Veldhoven, 2016), enabling a high degree of autonomy and control over work.

As mentioned in Section 2.1.1, physical aspects of a job can be qualified as job resources if they are functional in achieving work goals and/or stimulating personal growth, learning and development (Bakker and Xanthopoulou, 2013). Those physical aspects of job resources that potentially also foster autonomy and control over work, i.e., a remote workplace which is free of high levels of noise and distraction, and which is properly equipped with work devices, are covered by the concept telework workplace suitability. In suitable telework settings, employees benefit from stronger feelings of autonomy (Gajendran and Harrison, 2007), control, and flexibility (Huws et al., 1990; Standen et al., 1999; Maruyama and Tietze, 2012), which is why (a) preventing distractions and (b) enabling concentration are the main aspects of telework workplace suitability besides (c) suitable equipment for executing work tasks effectively.

#### 2.1.3. Collaboration

Teleworkers fear becoming isolated from the information network with colleagues, supervisors, and customers as a result of physical and temporal separation (Cooper and Kurland, 2002; Tremblay, 2003; Kossek et al., 2006; Lautsch et al., 2009). Many teleworkers rely on the assumption that “face time”, or visibility, at a central location is considered to be essential for outstanding performance evaluations (O‘Mahony and Barley, 1999). Performance evaluations and feedback are usually critical to career success (Gajendran and Harrison, 2007). The lack of face-to-face interaction with colleagues represents one of the main differences between office working and teleworking during COVID-19 (Toscano and Zappalà, 2020). As a result, experiences during the pandemic have shed new light on the threat of losing collaboration, since employees had to work remotely for long periods of time.

Work collaboration not only determines the ability of workers to solve problems and to cooperate with colleagues (Bar-On, 1995), but also to establish friendly relationships, helping them to accomplish their tasks (Hsu, 2017). Following Jassawalla and Sashittal (1998, p. 239), collaboration is “the coming together of diverse interests and people to achieve a common purpose via interactions, information sharing, and coordination of activities”. Collaboration is a social behavior that is crucial in the workplace whenever tasks are interdependent (Friedman et al., 2018). Thus, it is a key mechanism through which employees develop solutions to work-related problems with the support of their coworkers (Gruenfeld et al., 1996; Hsu, 2017).

#### 2.1.4. Work performance

Job resources such as social support, performance feedback, and autonomy have motivational potential and lead to high work engagement, low cynicism, and excellent performance (Bakker and Demerouti, 2007). Telework indirectly influences work performance by raising perceptions of control over the location and timing, and in terms of completing one's work (Gajendran and Harrison, 2007). Moreover, the environment in which people work affects their job performance (Davis, 1984). Therefore, if telework succeeds in covering these aspects of job resources, it has the potential to improve work performance and to reduce staff turnover (Igbaria and Guimaraes, 1999; Staples et al., 1999; Bailey and Kurland, 2002).

### 2.2. Hypotheses development

In a compulsory telework setting, organizational and social support is pivotal to successfully managing the boundary between work and personal roles, which is one of the key issues related to telework (Golden et al., 2006). If telework tasks are suitable, it is likely that teleworkers will stay connected with their stakeholders and, thus can obtain the necessary social and organizational support, which is particularly important as a negativity buffer in helping teleworkers to cope with stress and to remain focused on tasks (Bavik et al., 2020; Wang et al., 2020). Perceived proximity can be facilitated by allowing teleworkers to communicate frequently and by sharing personal information with remote colleagues to help identify personal similarities and to develop stronger relationships (O'Leary et al., 2014). When connecting employees with similar interests and enabling them to coordinate to share knowledge, teleworkers can learn from the experiences of coworkers, thereby strengthening collaboration (Mathieu et al., 2000; Kwahk and Park, 2016). Suitable telework tasks facilitate the use of digital communication tools, e.g., enterprise social media, to enable virtual meetings for consultation, projects and assignments, and thus enable continual coordination with colleagues and supervisors. Against this background, telework tasks suitability enables remote workers to collaborate and exchange effectively, which is why we hypothesize the following:

*H*_1_: Telework tasks suitability has a positive relationship with collaboration.

Job resources such as social support, performance feedback, opportunity for professional development, and autonomy refer to the physical, psychological, social, and organizational aspects of the job that are functional in achieving work goals or stimulating personal growth, learning, and development (Bakker and Demerouti, 2007; Bakker, 2011). They have motivational potential and lead to high work engagement, low cynicism, and excellent performance (Bakker and Demerouti, 2007). Telework tasks suitability covers these job resources. Therefore, we hypothesize the following:

*H*_2_: Telework tasks suitability has a positive relationship with work performance.

Many employees had to suddenly start working remotely from one day to the next during the pandemic. Therefore, there was often a lack of physical and digital infrastructure at the telework workplace. In an inappropriate remote working place, employees struggle with their ability to make decisions and to exercise a degree of discretion over the work to be accomplished; hence, they lose control of their work (Hackman and Oldham, 1980; Spector, 1986). Telework workplace suitability represents, among others, the job resource autonomy. As mentioned previously, the need for autonomy is a fundamental employee motivator (Gajendran and Harrison, 2007; Deci et al., 2017; Caligiuri et al., 2020). Experiencing fewer distractions helps to promote autonomy and control of work, which is why teleworkers are more likely to collaborate and establish a friendly relationship; a coming together of diverse interests and people to achieve a common purpose via interactions (Jassawalla and Sashittal, 1998). Therefore, we hypothesize the following:

*H*_3_: Telework workplace suitability has a positive relationship with collaboration.

Remote work has been linked to improved productivity (Harker and MacDonnell, 2012; Allen et al., 2015) if the home offers a quieter workplace where employees may be able to focus on their tasks better, away from interruptions and the many possible distractions of the traditional workplace (Anderson and Kelliher, 2020). To perform their telework tasks effectively, employees demand an environment in which they can concentrate and do not experience distractions, e.g., by household members (Bailey and Kurland, 2002; Haddad et al., 2009), since fewer distractions can lead to more goal progress, i.e., work performance (Brunstein, 1993). More specifically, if employees do not experience stress, work performance is among the most commonly cited positive outcomes of telework (Kossek et al., 2006; Siha and Monroe, 2006; Khanna and New, 2008). Telework workplace suitability covers autonomy, which leads to excellent performance (Bakker and Demerouti, 2007), since it raises perceptions of control over the location and timing, and in terms of completing one's work (Gajendran and Harrison, 2007). Against this background, we hypothesize the following:

*H*_4_: Telework workplace suitability has a positive relationship with work performance.

Teleworkers who do not collaborate due to feelings of isolation are less likely to receive, accurately interpret, or use important information, which will adversely affect their job performance (Rook, 1984), their interpretation of situations in social and political structures (Kurland and Terri, 1999), and their ability to launch valued job initiatives (Mann et al., 2000). However, when employees experience a low level of isolation and thus collaborate effectively, high work performance is among the most commonly cited positive outcome of teleworking (Siha and Monroe, 2006; Khanna and New, 2008). Additionally, numerous empirical studies have shown a significant positive relationship between collaboration and work performance (e.g., Wageman and Baker, 1997; Van der Vegt and Van de Vliert, 2016). Against this background, we propose the following:

*H*_5_: Collaboration has a positive relationship with work performance.

Furthermore, we expect an indirect effect of telework tasks suitability and telework workplace suitability on work performance. This is motivated by the observation that collaboration succeeds in establishing friendly relationships between teleworkers and their colleagues, enabling them to accomplish their tasks (Hsu, 2017), and hence, to achieve a certain level of work performance. Moreover, to manage the tasks successfully when teleworking, sound equipment (e.g., a notebook) is necessary to use enterprise social media, which improves work performance through knowledge sharing, collaboration, and communication visibility (Fulk and Yuan, 2013; Ellison et al., 2015). It is widely used by employees in the workplace to interact and collaborate with each other (Uysal, 2016; He et al., 2017) to perform work adequately.

## 3. Materials and methods

### 3.1. Sample and data

The central aim of this study is to examine the relationship between telework tasks and workplace suitability and collaboration and work performance in the context of a rapid transition from office work to telework. To assess our conceptual model shown in Figure 1 including its hypotheses, we conducted a survey. To test our hypotheses, we required a sample of workers who had experienced a rapid transition from office to remote work. Ideally, the majority of such employees had worked (mostly) in the office pre-COVID-19 and (mostly) remotely during the pandemic. If so, the employees' perceptions and evaluations of the sudden transition and its adherent switch in communication infrastructure would be reflected in the survey results. To meet the needs of this study, we selected a corporation that underwent a major transition from office work to remote work. The headquarters of this internationally operating manufacturing company with 14,000 employees is located in Germany. In collaboration with the corporation's management board, we identified 1,000 employees in Germany who were considered as office workers.

The questionnaire was developed and extensively discussed with the corporation's human resource (HR) department, i.e., with the senior business partner corporate HR and the head of corporate HR. Next, the questionnaire was presented by the third author to members of the corporate management board, i.e., the deputy spokesman of the board, the director for marketing, communication and politics, the technology director, the director of the operation's business segment, and the director of finance. Moreover, it was pre-tested with ten employees of the HR department to rule out technical issues. In the survey itself, we sent the questionnaire to 1,000 employees and received 848 responses. After removing respondents who had never worked at home during the COVID-19 pandemic (*n* = 109) and removing responses containing missing values (*n* = 48), we obtained a sample of 691 responses for our final analysis. Most of the respondents were men (53.98%). Supervisors accounted for 22% of all respondents. Before the pandemic, 75.40% of the respondents for our final analysis had never worked remotely for this employer. Table 1 presents the respondents' characteristics.

**Table 1:** Respondent characteristics.

**Characteristic**	***n* (%)**	**Characteristic**	***n* (%)**
Gender		Age (years)	
Female	317 (45.88)	29 or younger	188 (27.21)
Male	373 (53.98)	30-39	189 (27.35)
Diverse	1 (0.14)	40-49	146 (21.13)
		50 or older	168 (24.31)
Job position		Seniority (years)	
Supervisor	150 (21.70)	Less than 2	132 (19.10)
Staff member	510 (73.81)	More than 2, less than 5	194 (28.08)
Apprentice, trainee, or student	19 (2.75)	More than 5, less than 10	113 (16.35)
Other	12 (1.74)	More than 10	252 (36.47)
Frequency of working remotely before COVID-19 (days per week)		Frequency of working remotely during COVID-19 (days per week)	
1 or less	93 (13.45)	1 or less	87 (12.59)
1-2	42 (6.08)	1-2	128 (18.52)
2-3	19 (2.75)	2-3	194 (28.08)
3 or more	16 (2.32)	3 or more	282 (40.81)
Never	521 (75.40)	

### 3.2. Concepts and their operationalization

In our study, we deal with two types of concepts: formed concepts and behavioral concepts (Edwards, 2001; Henseler, 2017; Schuberth et al., 2018; Benitez et al., 2020; Henseler and Schuberth, 2020a; Hubona et al., 2021; Schuberth, 2021)^1^. In brief, formed concepts are the abstractions of design science, defined by a set of components and can be operationalized with help of a composite model (Henseler et al., 2015; Hubona et al., 2021; Yu et al., 2021), in which the concept is represented by an emergent variable (Henseler and Schuberth, 2020b). Accordingly, an emergent variable is completely determined as a weighted sum of components (Henseler and Schuberth, 2020b). It should be noted that an emergent variable in the composite model is not just a composite, i.e., a linear combination of (observed) variables, but is also a composite in which the observed variables act along a single dimension (Dijkstra, 2017). In contrast, behavioral concepts are regarded as ontological entities that are assumed to exist in nature (Borsboom, 2008) and enter the discipline's knowledge base through discovery. They are predominantly operationalized with help of reflective measurement, in which a latent variable represents the concept (Bollen and Bauldry, 2011).

The concepts telework tasks suitability and telework workplace suitability follow the notion of design research. Design research follows a pragmatist paradigm. Hence, in design science, concepts are the outcome of human development; they come into being through invention, are context-specific, and inextricably linked to purposefulness, i.e., teleology (Horvath, 2004; Møller et al., 2009; Baskerville and Pries-Heje, 2010; Henseler and Schuberth, 2020a). This makes design concepts conceptually equal to artifacts, the central phenomena of design science (Simon, 1969). Design science focuses on a future world and asks questions such as “How can …?” and “How should …?” (Henseler and Guerreiro, 2020). It can be seen as a quest for understanding and improving human performance (van Aken, 2005), “emerg[ing] from ongoing social and economic practices” (Orlikowski and Iacono, 2001, p. 131). The scientific inquiry of design concepts should mainly engage in synthesis rather than analysis, since a design concept must be complete, based on its items, which are its ingredients.

#### 3.2.1. Telework tasks suitability

In our study, telework tasks suitability is understood as a formed concept. It covers the following job resources: autonomy, social support, performance feedback, and opportunities for professional development. Therefore, it is defined by the following six components; “Which of the following assignments are suitable to be done at home easily?” (rated from 0: not suitable at all to 5: very suitable): (1) contacts with contact persons and customers; (2) coordination and consultation with supervisors; (3) assignments concerning projects, concepts and new topics, and the obligatory (4) virtual meetings (for projects and detailed assignments); (5) daily routines and (6) virtual meetings (for consultation). Consequently, this concept was operationalized in terms of the composite model and therefore modeled as an emergent variable.

#### 3.2.2. Telework workplace suitability

Similarly, telework workplace suitability is regarded as a formed concept. It covers the job resource autonomy and the need for an undisturbed remote workplace. In the following, we postulate that this concept is partly defined by the following two components; “What do you think about the following statements about work from home?” (rated from 0: strongly disagree to 5: strongly agree): (1) at home, it is easier to concentrate and stick to my assignments, and (2) at home, I get more distracted by third than I do in the office (reverse coded). Moreover, we suggest that specific work equipment is required for telework workplace suitability. Therefore, we asked respondents “What devices/hardware did your employer provide you with to work remotely?”: (1) notebook, (2) tablet, (3) mobile phone, (4) software for virtual meetings, (5) headset, and (6) printer. Finally, we assume that these eight components determine telework workplace suitability.

#### 3.2.3. Collaboration

Collaboration can help prevent teleworkers from feeling isolated. It can be regarded as a behavioral concept, which is why collaboration is modeled as a latent variable in the reflective measurement model. To measure collaboration, we used the following three items (which were rated from 1: is getting very bad to 5: is getting much better): (1) How does working from home affect collaboration with team members? (2) How does working from home affect your individual intervention with colleagues? (3) I miss the personal contact with my colleagues (reverse coded).

#### 3.2.4. Work performance

The ideal way to measure work performance would be in terms of objective performance-based assessment. However, due to the enormous variation in coverage and sophistication, using such an approach in a broader context is impossible (Kessler et al., 2003). As emphasized by Kessler et al. (2003, p. 159) “workers are in a better position than researchers to recognize the work performance domains that are most relevant to their particular occupations, to evaluate their recent performance in these domains, and to arrive at a rating of their overall work performance based on this evaluation”. Therefore, and in line with Toscano and Zappalà (2020), who investigated the role of concerns about COVID-19 among employees, we measured work performance by including a single-item scale in the questionnaire about individual perceptions of work performance when working remotely. The sample correlation matrix of the observed variables used in the final analysis is shown in the Appendix.

### 3.3. Test of common method variance

To reduce the potential effects of common method variance (CMV), we applied various procedural techniques. Specifically, we guaranteed confidentiality and anonymity in the survey administration; respondents were not allowed to return to previous questions, and questions were not tagged and were not given in the order hypothesized in the model (Podsakoff et al., 2003). Moreover, we carefully constructed the survey items by aiming to include unambiguous and concise questions (Tourangeau et al., 2000), and respondents completed the questionnaire voluntarily.

In addition to procedural techniques, we assessed statistically the potential effect of CMV. In doing so, we conducted Harman's single factor test. The total variance explained by a single factor is 15.88% which falls below the recommended threshold of 50% (Dupuis et al., 2020). As the effectiveness of Harman's single factor test has been criticized (Podsakoff et al., 2003), we also applied the measured latent marker variable (MLMV) approach as proposed by Chin et al. (2013). We decided to use construct level correlation (CLC) and included a CMV control construct (Chin et al., 2013). Specifically, we included the concept “Trust” as single-indicator control construct measured by the following 6-point scale item ranging from 0 (strongly disagree) to 5 (strongly agree): “I like that my employer trusts me in working remotely”. The CMV construct was modeled as antecedent of the latent variables, i.e., collaboration and work performance. As expected, trust showed only little impact on the latent variables collaboration (β = 0.020) and work performance (β = 0.017). Moreover, the results revealed that the path coefficients of our original model did not substantially change after the inclusion of the marker variables, i.e., no path coefficient (β) changed by more than 0.005. As a consequence, all these tests taken together suggest that potential CMV does not substantially undermine the results of this study. Therefore, the CMV construct was removed before the final analysis.

### 3.4. Model estimation

**Figure 2:**
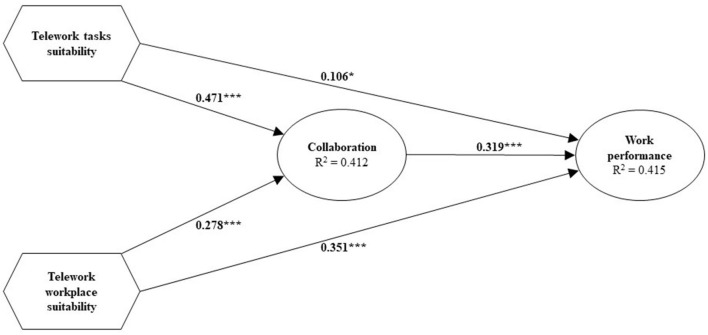
Results of PLS estimation. *p < 0.05, **p < 0.01, and ***p < 0.001.

The proposed research model was estimated using partial least squares path modeling (PLS-PM) as implemented in the cSEM R package (Rademaker and Schuberth, 2020). In its current form, known as consistent partial least squares (PLSc, Dijkstra and Henseler, 2015b), PLS-PM can be used for confirmatory research that deals with both latent and emergent variables (Müller et al., 2018; Benitez et al., 2020), which is the case for our study. Specifically, for the concepts modeled as emergent variables PLS-PM mode B was employed, i.e., PLS-PM mode B weights were used to form the emergent variables, while for the concepts modeled as latent variables PLS-PM mode A was combined with a correction for attenuation. Moreover, to draw statistical inference about the parameters, we rely on percentile bootstrap confidence intervals (CIs) based on 999 bootstrap runs (Aguirre-Urreta and Rönkkö, 2018). Before model estimation, we ensured that the specified model was identified, i.e., a unique solution existed for the model parameters. Since PLS-PM, including PLSc, builds on composites, irrespective of whether a concept is modeled as a latent or emergent variable, the identification rules for composite models needed to be applied (Dijkstra, 2017; Henseler and Schuberth, 2020b; Yu et al., 2021): (1) To fix the scale of each composite, the weights were scaled to ensure that each composite had a unit variance, which is the default setting in PLS-PM. Since this way of fixing the scale does not determine the sign of the weights, we applied the dominant indicator approach (Henseler et al., 2016), i.e., determined one observed variable per construct that must correlate with the construct positively. The dominant indicators were chosen as follows: “Virtual meetings (for consultation)” for telework tasks suitability, “At home, it is easier to concentrate and stick to my assignments” for telework workplace suitability, and “How does working from home affect collaboration with team members?” for collaboration. (2) As shown in Figure 2, no construct is isolated in the structural model. (3) Our structural model is recursive without correlated disturbance terms. As a consequence, all model parameters are identified^2^.

## 4. Results

To validate our proposed model, we followed the recently proposed guidelines of Benitez et al. (2020). Hence, as a first step, we assessed overall model fit. The bootstrap-based test for overall model fit indicates a misfit of the model (squared Euclidean distance: *d*_*L*_ = 0.346; *p* < 0.01; geodesic distance: *d*_G_ = 0.082; *p* < 0.01). However, it is well-known that for larger sample sizes, such as ours, the test for exact model fit is highly sensitive. Therefore, we also considered various fit indices that have been proposed in the PLS context (Schuberth et al., 2022). All fit indices indicate that our model fits the data reasonably well (Schumacker and Lomax, 1996; Marsh and Grayson, 2002; Schermelleh-Engel et al., 2003): standardized root mean square residual (SRMR) = 0.045, normed fit index (NFI) = 0.914 and goodness-of-fit index (GFI) = 0.943. As a next step, we focused on the composite and reflective measurement models.

Composite models were estimated by using mode B in PLS-PM; i.e., regression weights were used to form the emergent variables, and therefore collinearity among indicators forming an emergent variable was investigated in terms of the variance inflation factor (VIF). For the two composite models, the VIF values of the weights ranged from 1.043 to 3.038, suggesting that multicollinearity is not a problem (Hair et al., 2011). All weight and composite loading estimates of telework tasks suitability show the expected sign and are significant at a 5% significance level except for one, i.e., the estimated weight of coordination and consultation with supervisors. Considering telework workplace suitability, only the weights of “At home, it is easier to concentrate and stick to my assignments,” “At home, I get more distracted by third than I do in the office” and “Notebook” are significant at a 5% significance level. Following Benitez et al. (2020), we took a more conservative stance and decided not to remove components that show a non-significant weight and loading to preserve the constructs' content validity.

**Figure 3:**
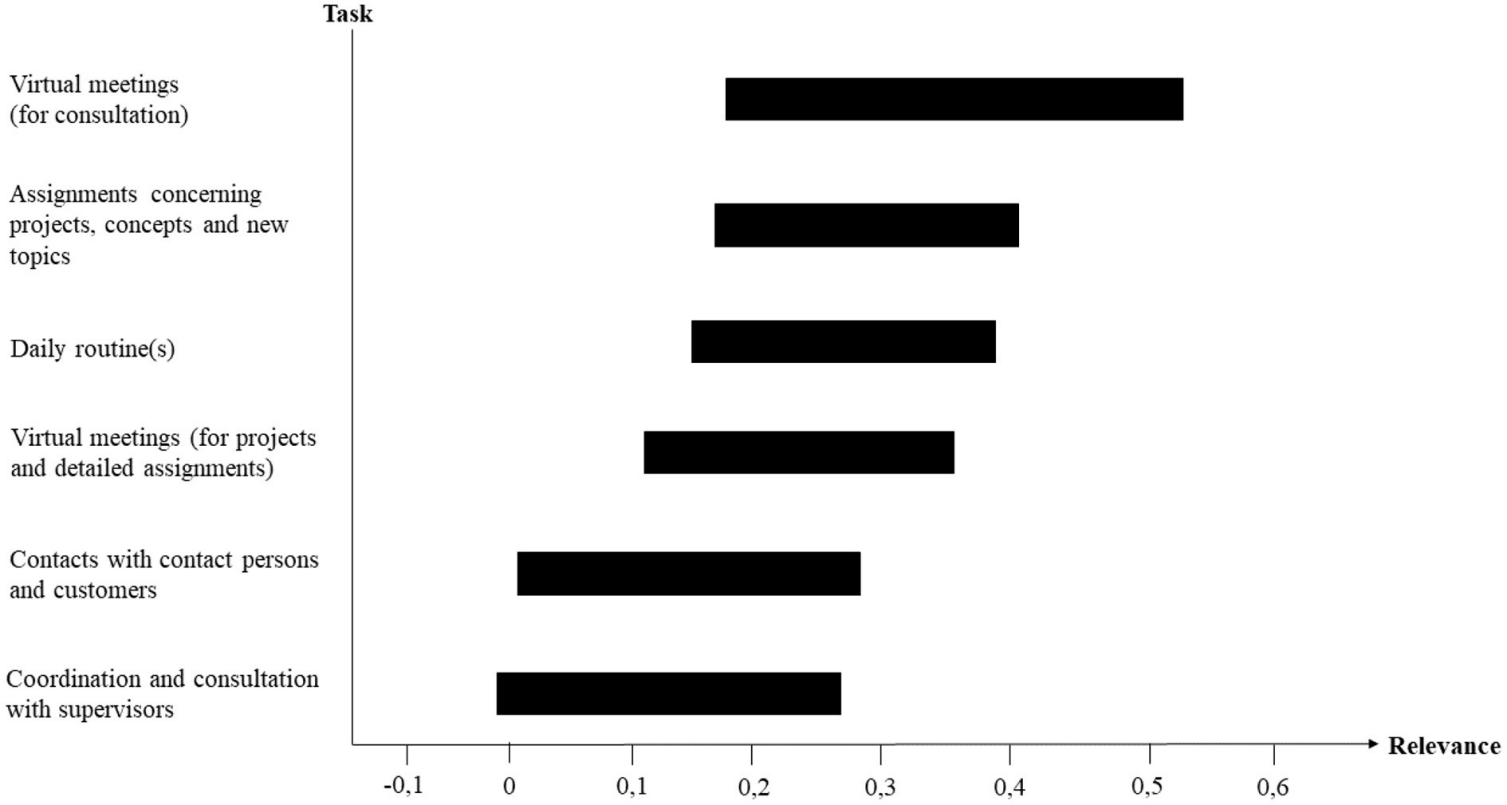
Relevance of tasks that form telework tasks suitability.

Figure 3 shows the relevance of each component in determining telework tasks suitability, i.e., the 95% percentile bootstrap confidence intervals of the weights are illustrated. The most relevant ingredient for telework tasks suitability is “Virtual meetings (for consultation)”, followed by “Assignments concerning projects, concepts and new topics” and “Daily routine(s)”. Furthermore, “Virtual meetings (for projects and detailed assignments)” are less relevant than “Virtual meetings (for consultation)”. The components “Contacts with contact persons and customers” and “Coordination and consultation with supervisors” appear to play the smallest roles.

**Figure 4:**
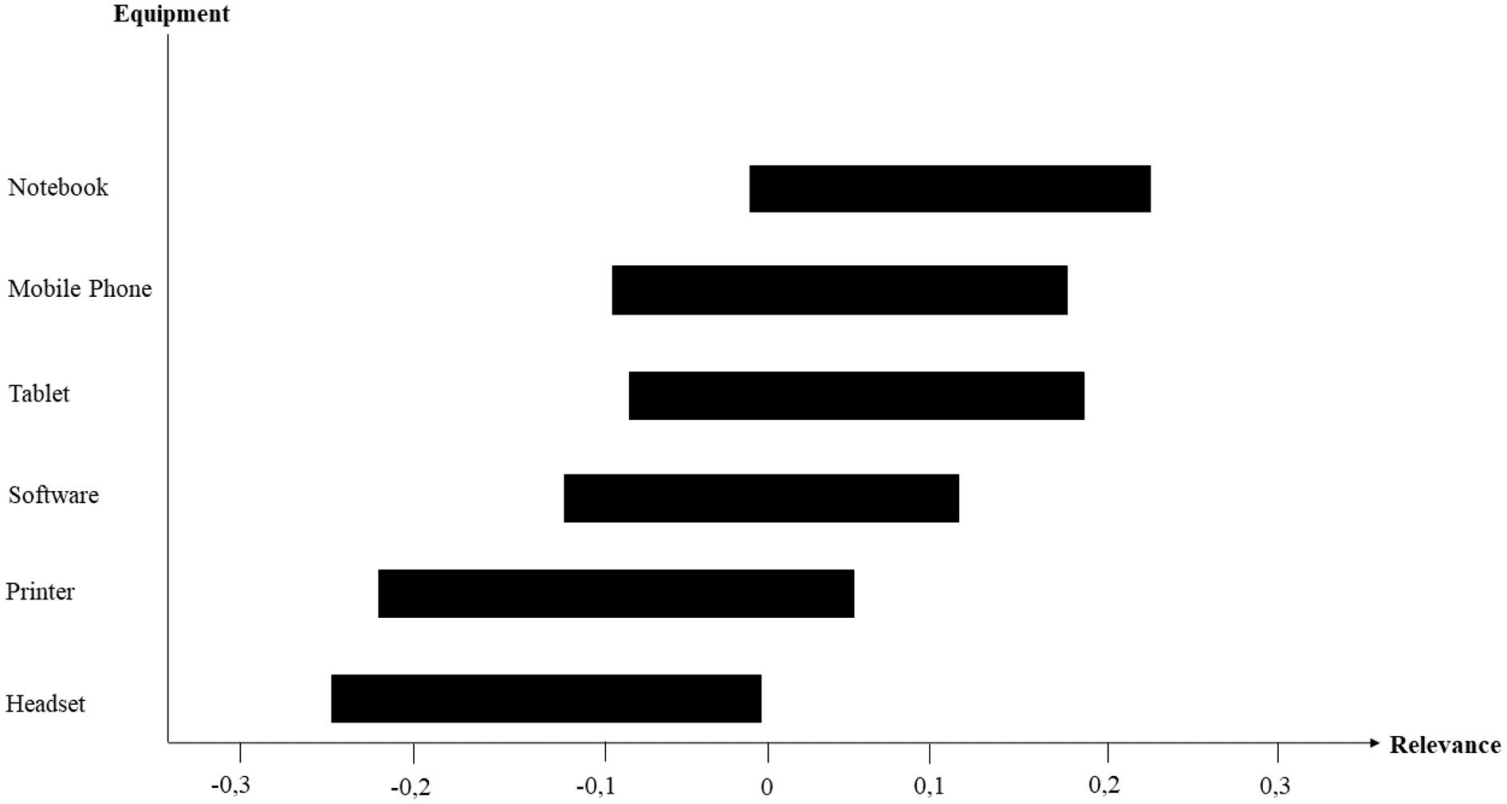
Relevance of equipment that partially forms telework workplace suitability.

To assist managers with the process of finding ingredients for telework workplace suitability, respondents were asked “What devices/hardware did your employer provide you with to work remotely?” Figure 4 shows the relevance of the equipment that partially determines telework workplace suitability, i.e., the 95% percentile bootstrap confidence intervals of the equipment weights are displayed. The most relevant work equipment that teleworkers have been provided with are notebooks. Whereas the provision of a mobile phone, a tablet, and software can also be beneficial for teleworkers to fulfill tasks properly, printers and headsets appear to play only a minor role.

Considering the reflective measurement model, we assessed the composite reliability in terms of ρ_*A*_ (Dijkstra and Henseler, 2015a). The ρ_*A*_ value for collaboration was 0.740 and thus above the recommended reliability threshold of 0.7 (Nunnally and Bernstein, 1994), indicating reliable construct scores. As a next step, we assessed convergent validity in terms of the average variance extracted (AVE; Fornell and Cha, 1994). For collaboration, the AVE was 0.483 and thus very close to the recommended threshold of 0.5 (Fornell and Larcker, 1981). Moreover, to assess discriminant validity for the latent variables, we applied the heterotrait-monotrait ratio of correlations (HTMT, Henseler et al., 2015) and its recently proposed enhancement, namely, the HTMT2 (Roemer et al., 2021), which allows for the assessment of discriminant validity in the context of congeneric measurement models. The HTMT and HTMT2 between collaboration and work performance both were 0.548, and thus clearly below the recommended threshold of 0.85 (and of 0.90). In addition, the one-sided 95% percentile bootstrap CIs of the HTMT (0.604) and the HTMT2 (0.605) did not cover 1; i.e., they were significantly different from 1, indicating no violation of discriminant validity. Finally, Table 2 shows that all standardized factor loading estimates are significant and range from 0.635 to 0.745, indicating indicator reliability ranging from 0.404 to 0.554. Table 3 reports the construct correlations. Additionally, Table 4 presents the direct, indirect and total effects of the variables of interest.

**Table 2:** Key concepts and their indicators.

**Constructs and indicators**	**VIF**	**Weight**	**95% CI**	**Loading**	**95% CI**
**Telework tasks suitability** (emergent variable, mode B)					
(variables rated from 0 = not suitable at all to 5 = very suitable)
Contacts with contact persons and customers.	1.350	0.160^*^	[0.032; 0.288]	0.605^***^	[0.499; 0.700]
Coordination and consultation with supervisors.	3.038	0.106	[-0.076; 0.265]	0.734^***^	[0.641; 0.811]
Assignments concerning projects, concepts and new topics.	1.521	0.296^***^	[0.170; 0.409]	0.732^***^	[0.643; 0.796]
Virtual meetings (for projects and detailed assignments).	1.503	0.238^***^	[0.111; 0.367]	0.677^***^	[0.585; 0.757]
Daily routine(s).	1.131	0.278^***^	[0.155; 0.392]	0.573^***^	[0.458; 0.665]
Virtual meetings (for consultation).	2.943	0.371^***^	[0.179; 0.534]	0.777^***^	[0.684; 0.844]
**Telework workplace suitability** (emergent variable, mode B)				
(variables rated from 0 = strongly disagree to 5 = strongly agree)
At home, it is easier to concentrate and stick to my assignments.	1.123	0.816^***^	[0.720; 0.892]	0.931^***^	[0.867; 0.962]
At home, I get more distracted by third than I do in the office. (reverse coded)	1.134	0.303^***^	[0.163; 0.416]	0.566^***^	[0.433; 0.658]
***Equipment provided by the employer***					
(measures: 1 = yes, 0 = no)				
Notebook	1.198	0.115^*^	[0.005; 0.225]	0.220^***^	[0.098; 0.334]
Tablet	1.419	0.048	[–0.080; 0.176]	0.147^*^	[0.019; 0.278]
Mobile phone	1.566	0.044	[–0.097; 0.188]	0.131	[0.001; 0.266]
Software for virtual meetings	1.118	–0.004	[–0.123; 0.114]	0.002	[–0.133; 0.141]
Headset	1.060	–0.148	[–0.251;–0.040]	–0.163	[–0.282;–0.038]
Printer	1.043	-0.090	[-0.215; 0.047]	–0.077	[–0.244; 0.097]
**Collaboration** (latent variable, mode A consistent)					
(variables rated from 1 = is getting very bad to 5 = is getting much better)					
How does working from home affect collaboration with team members?		0.442^***^	[0.404; 0.480]	0.745	[0.672; 0.806]
How does working from home affect your individual intervention with colleagues?		0.377^***^	[0.345; 0.410]	0.635	[0.566; 0.705]
I miss the personal contact with my colleagues. (reverse coded)		0.416^***^	[0.383; 0.450]	0.700	[0.635; 0.762]
**Work performance** (single-indicator construct)					
(variables rated from 1 = is getting very bad to 5 = is getting much better)				
How does working from home affect your individual work performance?		1.000		1.000	

CI = Percentile bootstrap confidence interval;

* *p* < 0.05,

** *p* < 0.01, and

*** *p* < 0.001.

**Table 3:** Construct correlation matrix.

**Construct**	**Collaboration**	**Telework tasks suitability**	**Telework workplace suitability**	**Work performance**
Collaboration	1.0000	
Telework tasks suitability	0.5908	1.0000
Telework workplace suitability	0.4807	0.4898	1.0000
Work performance	0.5500	0.4450	0.5495	1.0000

**Table 4:** Evaluation of the structural model.

**Relationship**	**Direct/Indirecteffect**	**95% CI**	**f^2^**	**Totaleffect**	
Telework tasks suitability → Collaboration	0.471	[0.400; 0.548]	0.308	0.471	H1: Supported
Telework tasks suitability → Work performance	0.106	[0.016; 0.196]	0.012	0.256	H2: Supported
Telework workplace suitability → Collaboration	0.278	[0.207; 0.361]	0.107	0.278	H3: Supported
Telework workplace suitability → Work performance	0.351	[0.280; 0.426]	0.155	0.439	H4: Supported
Collaboration → Work performance	0.319	[0.219; 0.418]	0.102	0.319	H5: Supported
Telework tasks suitability → Collaboration → Work performance	0.150	[0.098; 0.214]	—	0.256	
Telework workplace suitability → Collaboration → Work performance	0.089	[0.057; 0.132]	—	0.439	

CI = Percentile bootstrap confidence interval.

Considering our hypotheses, we found support for H1, H2, H3, H4, and H5, indicating that telework tasks suitability has a positive relationship with collaboration (H1) (β = 0.471; 95% CI [0.400 to 0.548]; *f*^2^ = 0.308) and has a positive relationship with work performance (H2) (β = 0.106; 95% CI [0.016 to 0.196]; *f*^2^ = 0.012). The *f*^2^ value indicates a medium effect of the relationship between telework tasks suitability and collaboration. Telework workplace suitability has a positive relationship with collaboration (H3) (β = 0.278; 95% CI [0.207 to 0.361]; *f*^2^ = 0.107) and with work performance (H4) (β = 0.351; 95% CI [0.280 to 0.426]; *f*^2^ = 0.155). Considering the *f*^2^, the latter effect is medium, while the former effect is weak. Additionally, collaboration has a positive relationship with work performance (H5) (β = 0.319; 95% CI [0.219 to 0.418]; *f*^2^ = 0.102). However, the *f*^2^ indicates only a weak effect. As the results of the mediation analysis revealed, collaboration positively mediates the relationship between telework tasks suitability and work performance (β = 0.150; 95% CI [0.098 to 0.214]). The indirect effect is larger than the direct effect, indicating a complementary mediation (Zhao et al., 2010). Also, telework workplace suitability has a positive relationship with work performance through the mediating variable collaboration. The direct effect of telework workplace suitability on work performance is larger than the indirect effect (β = 0.089; 95% CI [0.057 to 0.132]).

## 5. Discussions and conclusions

In our study, we investigated the relationships between telework tasks suitability, telework workplace suitability, collaboration, and work performance in the situation where office workers were made to telework due to the COVID-19 outbreak. The purpose was to gain insights into the degree of collaboration and work performance of inexperienced and involuntary teleworkers based on telework tasks suitability and telework workplace suitability. Drawn from the JD-R model, we theorized that telework tasks suitability and telework workplace suitability have a positive relationship with work performance and collaboration as they cover relevant job resources, i.e., the need for autonomy, for social support, for performance feedback, for professional development, and for an undisturbed remote workplace. Responses from 691 professional and managerial workers from Germany constituted the data set for this study. The empirical analysis supported our proposed theory. Our research is the first to conceptualize and operationalize the formed concepts telework tasks suitability and telework workplace suitability and to link them to job resources and assessing their relationships with collaboration and work performance. To achieve collaboration and work performance, tasks should be suitable for remote working to enable autonomy by processing daily work routines, to generate social support, and to meet the need for professional growth and performance feedback, e.g., by assigning, conducting, and consulting about projects, concepts, and topics. Collaboration mediates the relationship between telework tasks suitability and work performance. Telework workplace suitability showed a direct relationship with work performance. Caligiuri et al. (2020, p. 707) had already recommended studying those who are working from home for the first time, as “this group would uniquely enable us to examine the cross-national, generational, functional, etc. differences in predicting employees' preferences for working from home in the future, post-pandemic”. We acted on their call, as 75.40% of the respondents for our final analysis had never worked remotely before the pandemic.

### 5.1. Theoretical implications

By connecting formed concepts to behavioral concepts, this study advances knowledge on remote work during the COVID-19 pandemic. Insights from working at home during COVID-19 can, beyond the immediate context of the pandemic, guide future research on the potential benefits and potential drawbacks of working remotely. First, it introduced the concept telework tasks suitability and provided ingredients for its formation. We argued that the concept should be built on three main pillars to cover relevant job resources such as autonomy, social support, performance feedback, and opportunities for professional development, i.e., enabling teleworkers to (1) stay connected to their stakeholders, (2) process their daily work routines, and (3) address their need for professional growth. This is one central contribution of this paper. Second, the study introduced the concept telework workplace suitability and the relevant ingredients to comprise it. We argued that there are two main pillars to that concept to cover the job resource autonomy, i.e., by (1) preventing teleworkers from being distracted, and (2) enabling them to concentrate. Additionally, we argued that the remote workplace should be equipped with certain physical/digital assets. It turned out that a notebook and a tablet are relevant essentials for the remote workplace, but that other devices and software are not relevant. This is a further central contribution of this paper. As a result, our research is first to conceptualize and operationalize these two formed concepts for assessing their relationships with collaboration and work performance. Third, this study has identified the relevance of ensuring collaboration between teleworkers and their stakeholders, i.e., colleagues, supervisors, and customers. With this study, we aimed to provide researchers with a starting point for further research. More specifically, when conducting research in this context, i.e., evaluating the impact of work design measures on employees' behavior, foremost, researchers should differentiate between formed concepts and behavioral concepts (Yu et al., 2021). That is, when researchers identify and model a formed concept, the items should be understood as its ingredients, and not as its measures. Against this background, it is a priori relevant to define the type of concept as either behavioral or formed, since treating a behavioral concept as formed or a formed concept as behavioral simply means confusing natural science and design science. If so confused, estimates of the model parameters can be severely biased (Sarstedt et al., 2016).

### 5.2. Practical implications

As highlighted in the Introduction, many employees have come to expect their employers to continue offering flexible work arrangements and are even prepared to quit their jobs if a return to permanent office work is expected when the pandemic is over. Consequently, managers are advised to maintain the new normal, e.g., by implementing tasks that are suitable to provide a generic telework infrastructure for all teleworkers to maintain well-being and work performance. Those who had to commute frequently and suffered from a worsening work-life balance due to child caring duties, etc. appreciate flexibility in a temporal and local contexts. To cover the aforementioned job resources, the process of defining telework tasks suitability should be conducted carefully and in line with human resource capabilities, since a recent study has shown that the overload or complexity of technologies needed to work remotely has adverse outcomes on employees' well-being (Molino et al., 2020). Therefore, managers should strive for a generic, easy-to-use digital remote working environment that ideally will enable all teleworkers to fulfill their usual, familiar work tasks. As training to employees on how to use (novel) technologies was usually absent after the sudden switch from office work to telework, managers should initially focus on even trivial applications that primarily enable social support and performance feedback to avoid isolation during remote working days without risking the overextension of workers. We consider our study to be particularly relevant for stakeholders involved in the sudden transition from office to remote working that we have described. In particular, companies that adopted remote working programs should define opportunities to increase the degree of collaboration, since a quality relationship with one's supervisor is likely to facilitate good performance (Bakker and Demerouti, 2007), and thus decrease the feeling of social isolation. In doing so, employers should strive for telework tasks suitability to enable teleworkers to stay connected to their stakeholders and to fulfill (most of) their work tasks successfully. Employers should develop procedures to use technical infrastructure, e.g., social channels for remote collaboration, and should address technical challenges that need feedback from colleagues or supervisors. In our view, the concepts telework tasks suitability and telework workplace suitability and their relationships with collaboration and work performance will not only turn out to be significant in the context of the pandemic, but also post COVID-19. Since collaborating remotely can significantly improve work performance, managers should wherever possible incorporate the learning of this study in their decision-making processes on the design of work tasks and of the workplace concerning employees who telework regularly.

### 5.3. Limitations and directions for future research

This study also has limitations. First, it uses data from an internationally operating manufacturing company with 14,000 employees but surveyed only employees working in Germany. For this reason, the results can only be generalized to firms of similar size with workers in Germany. Future research should explore whether our proposed theory is supported in different contexts, such as other countries or small and medium-sized firms. Second, our sample mainly consists of respondents who had been office workers before the pandemic, but experienced a rapid transition from office to telework during the pandemic. Accordingly, the results can only be generalized to firms in which employees were not specifically experienced in telework before the COVID-19 pandemic, and in which telework was not previously widely used practice. Third, we did not distinguish between different job positions in our analysis. Future research should differentiate between professional categories, since employees who share similar qualities with other members of the organization enjoy more pleasant interactions, stronger social integration, and greater interpersonal attraction (Riordan, 2000; Horwitz and Horwitz, 2007). Fourth, the data were collected in spring 2021, during the pandemic, when many employees were forced to telework and thus could not properly prepare for remote working. Future studies should investigate the relationships between telework tasks suitability, telework workplace suitability, collaboration, and work performance in situations where teleworkers experience a planned and prepared transition from office to remote working. Fifth, the concepts telework tasks suitability and telework workplace suitability were self-designed. Similarly, the scale to measure collaboration was self-designed. Therefore, we recommend testing their validity in future research, especially post COVID-19. Sixth, complementary mediation can be indicative of an omitted mediator (Zhao et al., 2010). Hence, future research is asked to provide explanations for variables that mediate the relationship between telework tasks suitability and work performance, and for variables that mediate the relationship between telework workplace suitability and work performance. Tasks are allocated to capital or labor, and new technologies not only increase the productivity of capital and labor at tasks they currently perform, but also impact the allocation of tasks to these factors of production (Acemoglu and Restrepo, 2019). An increase in digitalization and the rapid transition from office to remote work might demand new tasks that are related to more specialized functions in existing occupations and have the potential to generate a positive productivity effect (Acemoglu and Restrepo, 2019), i.e., positive work performance. In our study, we rely on familiar, usual telework tasks to ensure a high level of autonomy after the enforced transition from office to remote work. Future research is asked to investigate the implications of capital directly displacing workers from tasks that they previously performed (Acemoglu and Autor, 2011).

## Data availability statement

The raw data supporting the conclusions of this article will be made available by the authors, without undue reservation.

## Ethics statement

Ethical review and approval was not required for the study on human participants in accordance with the local legislation and institutional requirements. Written informed consent for participation was not required for this study in accordance with the national legislation and the institutional requirements.

## Author contributions

TM was responsible for the conceptualization, the literature review, the methodology, the investigation, and for drafting the document. FS contributed to the formal analysis, review, editing, and co-supervision of the document. MB contributed to the data collection for investigation. JH contributed to the positioning, the theorizing, and the methodology and supervised the research process. All authors contributed to the article and approved the submitted version.

## Conflict of interest

JH acknowledges a financial interest in the composite-based SEM software ADANCO and its distributor, Composite. The remaining authors declare that the research was conducted in the absence of any commercial or financial relationships that could be construed as a potential conflict of interest.

## Publisher's note

All claims expressed in this article are solely those of the authors and do not necessarily represent those of their affiliated organizations, or those of the publisher, the editors and the reviewers. Any product that may be evaluated in this article, or claim that may be made by its manufacturer, is not guaranteed or endorsed by the publisher.

## Footnotes

^1^In the literature, formed concepts are also labeled as forged concepts (Henseler and Schuberth, 2020a; Yu et al., 2021).

^2^In an additional analysis, we considered the control variables age, gender, job position, and seniority to our model, including their direct effects on the latent variables. The direct effects of the control variables were neither significant nor did they alter the conclusion. Therefore, we continued with the model without control variables.

## Appendix

**Table A1:** Empirical correlation matrix.

	**Contacts **	**Coordsp **	**Newtopics **	**Meetingsprojects **	**Routines **	**Meetingsconsult **	**Concentrate **	**Distraction **	**Notebook **	**Tablet **	**Mobile **	**Software **	**Headset **	**Printer **	**Collabo **	**Intervention **	**Misscontact **	**Perform **
Contacts	1.0000	
Coordsp	0.4128	1.0000
Newtopics	0.3786	0.3648	1.0000
Meetingsprojects	0.3869	0.3764	0.5148	1.0000
Routines	0.2068	0.2389	0.2721	0.2397	1.0000
Meetingsconsult	0.3747	0.8048	0.3745	0.3200	0.2668	1.0000
Concentrate	0.2836	0.2631	0.3191	0.2674	0.2498	0.2563	1.0000
Distraction	0.2489	0.1222	0.1996	0.1823	0.1878	0.1029	0.3092	1.0000
Notebook	–0.0174	0.0986	0.0667	0.0368	0.0801	0.0530	0.0936	0.0769	1.0000
Tablet	–0.1159	0.0220	–0.0344	0.0970	0.0453	–0.0171	0.0621	0.0701	0.2500	1.0000
Mobile	–0.1899	0.0346	–0.0918	0.0122	0.0575	0.0031	0.0248	0.0810	0.3853	0.5231	1.0000
Software	–0.1343	–0.0816	–0.0462	–0.0414	–0.0547	–0.0490	0.0272	–0.0630	0.1314	0.2040	0.2449	1.0000
Headset	–0.1176	–0.0838	–0.1173	–0.0769	–0.1028	–0.0998	–0.0288	–0.0177	0.1203	0.0722	0.1178	0.1955	1.0000
Printer	–0.0448	–0.0693	–0.0169	–0.0112	0.0496	–0.0601	–0.0244	0.0877	0.0691	0.1575	0.1009	0.0400	0.0925	1.0000
Collabo	0.2615	0.3782	0.3529	0.3061	0.1904	0.4194	0.2934	0.1773	0.1349	0.1183	0.1149	–0.0260	–0.1002	0.0220	1.0000
Intervention	0.1820	0.3444	0.2442	0.2232	0.1355	0.3607	0.2793	0.1887	0.1113	0.1515	0.0802	0.0332	–0.0528	–0.0535	0.5017	1.0000
Misscontact	0.2448	0.2993	0.2973	0.2569	0.2054	0.3208	0.3109	0.2184	0.1637	0.0948	0.0997	0.0598	0.0091	–0.0370	0.4254	0.5271	1.0000
Perform	0.2932	0.2715	0.3261	0.3216	0.3358	0.2775	0.5268	0.3062	0.0613	0.0140	0.0208	–0.0175	–0.0950	–0.0468	0.4020	0.3497	0.3930	1.0000
